# Short and Extra Short Dental Implants in Osseous Microvascular Free Flaps: A Retrospective Case Series

**DOI:** 10.3390/jpm14040384

**Published:** 2024-04-03

**Authors:** Barbora Hocková, Rastislav Slávik, Basel Azar, Jakub Stebel, Dušan Poruban, Estevam A. Bonfante, Rolf Ewers, Yu-Chi Cheng, Adam Stebel

**Affiliations:** 1 Department of Maxillofacial Surgery, F. D. Roosevelt University Hospital of Banská Bystrica, 974 01 Banská Bystrica, Slovakia; bhockova@nspbb.sk (B.H.); rslavik@nspbb.sk (R.S.); dporuban@nspbb.sk (D.P.); astebel@nspbb.sk (A.S.); 2 Department of Prosthodontics, Faculty of Medicine and Dentistry, Palacky University, 779 00 Olomouc, Czech Republic; baselazar1@hotmail.com; 3 Dentaris Praha Dental Clinic, Olšanská 7, 130 00 Prague, Czech Republic; 4 3S DENT Dental Clinic, Šancová Street, 831 04 Bratislava, Slovakia; jakub.stebel@3sdent.sk; 5 Department of Prosthodontics and Periodontology, Bauru School of Dentistry, University of Sao Paulo, Bauru 17012, SP, Brazil; 6 The University Hospital for Cranio-Maxillofacial and Oral Surgery, Waehringer Guertel 18-20, 1090 Vienna, Austria; rolf@cmf-vienna.com; 7 CMF Institute Vienna, Schumanngasse 15, 1180 Vienna, Austria; 8 Harvard School of Dental Medicine, 188 Longwood Ave, Boston, MA 02115, USA; jack_cheng@hsdm.harvard.edu

**Keywords:** short dental implants, microvascular free flap, prosthetic rehabilitation, osseous microvascular free tissue transfer

## Abstract

There is limited information regarding implant and prosthetic survival after osseous microvascular free flap (OMFF). This case series aims to describe the placement of short and extra short implants in osseous microvascular free flaps to support prostheses, and present an up to 40-month retrospective follow-up. Short and extra short dental implants were placed in six fibula free flaps (FFF) and in two microvascular deep circumflex iliac artery (DCIA) flaps. In total, 27 short and extra short dental implants have been placed into two different types of free flaps. Kaplan–Meyer (K-M) survival analyses were performed to evaluate the survival and success outcomes of implants and prostheses. Out of the eight patients reconstructed with free flap, five were rehabilitated with prostheses, one patient has a temporary prosthesis, and two patients are in the process of prosthetic rehabilitation. Twenty-seven implants were followed up for up to 40 months, and K-M analyses showed 100% implant survival probability (95% confidence interval: 100%), while the implant success probability was 91.0% (95% confidence interval: 68.6–97.7%). Short and extra short dental implants placed in OMFF presented high survival and success rates in a retrospective case series after up to 40 months.

## 1. Introduction

In patients with bone defects of the head and neck, surgical reconstruction using osseous microvascular free flaps (OMFF) are the gold standard treatment [1]. Bone defects may be reconstructed in the upper or lower jaws with different types of osseous free flaps taken from various body sites; e.g., fibula free flaps (FFF) [2], deep circumflex iliac artery (DCIA) flaps, and scapula flaps (SF) [3]. The surgical result and the patient’s postoperative quality of life depends on the dental prosthetic rehabilitation. It is well known that articulation, speech, and chewing are major components of long-term success in reconstructive head and neck surgery, and contribute to the overall improvement in a patient’s quality of life [4].

The rehabilitation of patients treated with OMFF using conventional complete or partial dentures, even when the reconstruction is ideal, may be difficult or impossible due to the lack of stability of the prostheses in the compromised oral environment [5]. An excellent solution for this group of patients is rehabilitation with dental implants inserted into free osseous flaps to provide retention and support for the prostheses [6]. According to Misch’s Contemporary Implant Dentistry [7], there are countless advantages associated with implant-supported prostheses: e.g., the overall health and psychological improvement achieved as a result of improved stability and retention of the removable prostheses, reduced size of the prostheses, improved phonetics, and improved occlusion. The cumulative rates of prostheses free of complications after 5 and 10 years were 29.3% and 8.6%, respectively [8]. Available data show high survival rates for short and extra-short implants, and an implant survival rate of 97.2% [9]. It has been reported that the different ossification processes that develop the fibula and the jawbones may affect dental implant survival [10]. Therefore, OMFF patients and surgeons benefit from a collaborative multi-team approach focused on improving the long-term functional outcomes of the reconstructions [11].

Dental implants have become an indispensable and established therapy in dentistry for the replacement of missing teeth in different clinical situations [12]. In maxillofacial surgery, dental implants can be used to retain a dental prosthesis as well as for retention of different types of obturators, eye socket prostheses, or anchoring elements.

However, even with great improvements in reconstructive surgical techniques, such as free vascularized bone flaps, ideal implant placements cannot be easily achieved because of the limited dimensions of the available host bone in many patients [13]. For example, in fibula free flaps, the main disadvantage is the limited vertical height of the harvested bone, which is 1.3–2.3 cm. Also, patients’ mandibles may differ considerably in height, especially in younger patients [14]. Numerous surgical options are now available to overcome the limitations of bone availability, allowing implants to be placed in more favorable sites, possibly improving aesthetic outcomes. Surgical interventions such as guided bone regeneration, onlay bone grafting, sinus floor elevation, distraction osteogenesis, transposition of the inferior alveolar nerve, and the of use zygomatic or tilted implants have been developed and implemented [15].

Because of its straightforwardness, Al-Johany’s proposed classification scheme for a dental implant’s length will be used throughout this manuscript. It indicates that extra-short implants are 6.0 mm or less, and that short implants are longer than 6.0 mm and less than 10.0 mm [16]. Increasingly, the literature shows similar survival rates for short implants compared to standard sized implants [17,18,19,20,21,22]. Evidence also shows that short implants can be used successfully in atrophic jaws, reducing the need for invasive, complex surgery, and treatment morbidity [17,20,21,23]. Overall, prospective studies now indicate similar survival and success rates for short and standard dental implants [24].

The ideal bone free flap selection depends on several interrelated factors, including the timing of reconstruction, the recipient site characteristics, the defect location, and the need for bone and soft tissue [25]. High and non-significantly-different survival rates have been reported in a network meta-analysis of 1513 patients receiving FFF, DCIA, scapula flaps, or an osteocutaneous radial forearm flap [26]. In a 10-year retrospective study of prostheses supported by implants placed in various osseous flaps, lower success was observed for fixed partial prostheses (93%) compared to removable partial prostheses (100%), although no difference was observed in survival between different flaps [27]. In contrast, different outcomes have been reported in another retrospective cohort, where 100% survival was reported for implants placed in scapula flaps, 83% in FFF, 80% in radial composite free flaps, and 76% in DCIA [28].

Based on recent publications regarding the long term survival of short dental implants [9], we predict good long-term results, even in osseous microvascular free flaps. This being said, further studies will be needed to confirm that short dental implants can provide the same results in patients with cancer [15]. The literature is limited regarding short dental implant placements in OMFF. This manuscript will evaluate the success and survival rates of short and extra-short dental implants placed in patients reconstructed with OMFF by presenting a series of cases with up to 40 months follow-up.

## 2. Materials and Methods

**Figure 1:**
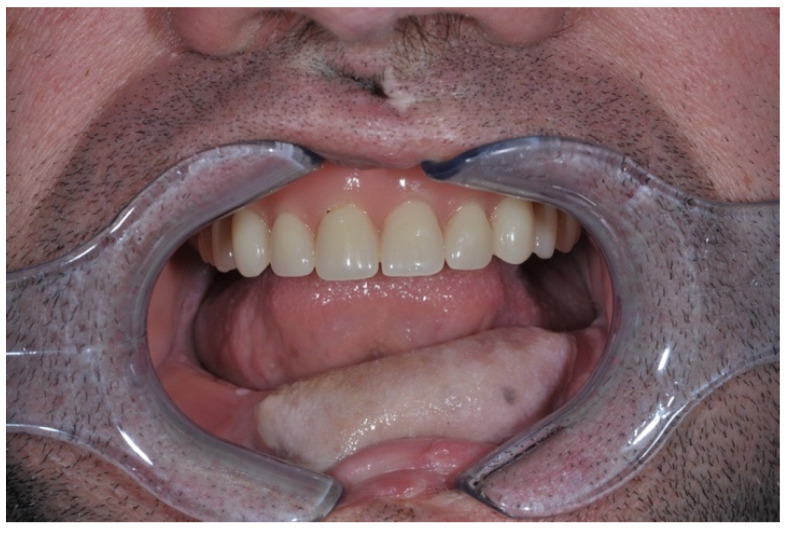
Clinical view of a patient with fibula free flap, and opposing arch with removable prosthesis.

Ethical approval was obtained from the Ethics Committee of F. D. Roosevelt Hospital (#17.1.2020). We have produced a list of criteria, which we followed, before selecting suitable patients for the placement of short and extra short dental implants in reconstructed jaws. One of the criteria involves the consideration of previous treatment. Therefore, in our protocol, two treatment options were available: (i) for patients who did not receive adjuvant oncological treatment or in cases where a microvascular bone flap was used for a secondary treatment, implant placement was delayed for a minimum of six months following jaw reconstruction; (ii) for patients with a history of adjuvant oncological treatment, we required a minimum delay of 12 months. Given that we inserted extra-short or short dental implants (Bicon LLC, Boston, MA, USA) into the OMFF, we determined that the minimum dimensions of an osseous free flap have a height of 6.0 mm and a width of 5.0 mm. Prosthetically, the occlusion of the opposing dentition has a significant impact on the rehabilitation (Figure 1). Before placing dental implants, every patient underwent consultation with a prosthodontist about possibilities of rehabilitation also in the opposite jaw.

A key point while placing dental implants was to place them subcrestally. During the postoperative period, we regularly confirmed the position of the bone segments with an orthopantomogram (OPG). Cone-beam computed tomography (CBCT) was routinely used for the placement of the implants. Extra- and intraoral photographs were taken on a regular basis. The condition of the opposing dentition and the patient’s hygiene were factors for inclusion in the study.

Dental implants (Bicon LLC, Boston, MA, USA) were placed in eight patients with microvascular free flaps. Preoperative CBCT and OPG were made for measurement of the bone before implantation. Subcrestal dental implant placement was performed according to the manufacturer’s instructions. The extra-short and short implant system used has a 1.5° locking taper implant–abutment connection, which provides 360° of universal abutment positioning [29]. The implant has a plateau-root form macro design with a calcium-phosphate-treated surface. The implant’s sloping shoulder provides sufficient space for bone to support the interproximal papillae, which are crucial for gingivally aesthetic restorations. Given the retrospective nature of the present case series cohort of diseased patients who were in need of vascularized free flaps and implants, there was no inclusion or exclusion criteria.

Demographic information of our sample was collected (Table 1). The group consisted of five men and three women with an average age of 43 years. The youngest patient was 21 years old and the oldest patient was 67 years old. Five patients had their mandible reconstructed and three patients had their maxilla reconstructed with OMFFs. Implants were placed in six fibula free flaps (FFF), and in two microvascular deep circumflex iliac artery flaps (DCIA). Three patients received reconstruction for malignant disease; one patient for osteoradionecrosis; two patients for traumatic injury; one patient for giant cell tumor; and one for an ameloblastoma associated with hypercalcemia. Four out of the eight patients received radiotherapy prior to placement of their implants. Twenty-seven implants were placed in two different types of OMFF. Five patients received an implant-supported prosthesis; one has a temporary one, and another two patients are in the process of prosthetic rehabilitation. One patient was rehabilitated using a millable fiber-reinforced hybrid composite framework, TRINIA^®^ (Bicon LLC, Boston, MA, USA), and composite resin teeth. Four patients were rehabilitated with a fixed metal ceramic prosthesis. Descriptions of two representative cases are provided below.

### 2.1. Patient 1—Case Report of Extra Short Dental Implants in Fibula Free Flap

**Figure 2:**
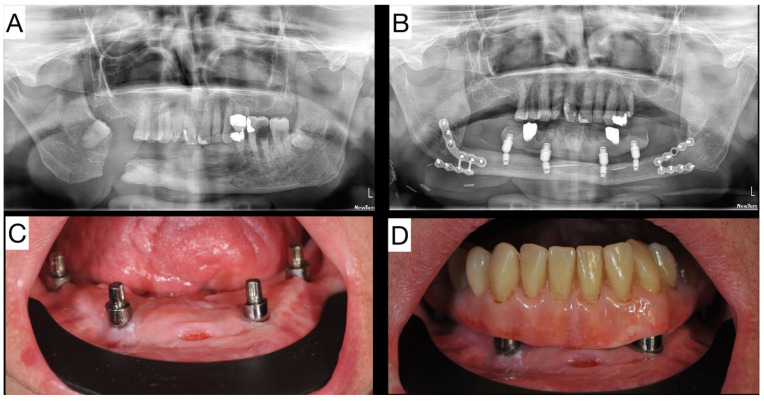
(**A**) Ortopanthomogram (OPG) of mandible after pathologic fracture due to osteoradionecrosis. (**B**) OPG of patient after angle to angle mandible resection and immediate reconstruction with a fibula free flap, and prosthetic rehabilitation supported by extra short dental implants at initial loading. (**C**) Intraoral image of four angled universal abutments on extra short implants in fibula free flap. (**D**) Prosthetic rehabilitation of mandible with a TRINIA® telescopic prosthesis.

The first patient was a 67-year-old male with previously diagnosed invasive squamous cell carcinoma, Grade 1, localized in the floor of the mouth. Patient underwent neck dissection on his right side in 2008 and resection of a carcinoma with free margins. After that, he underwent radiotherapy and developed osteoradionecrosis of the mandible, resulting in a pathologic fracture. He was first seen in October 2018 for the reconstruction of his mandible (Figure 2A). The patient had a chronic, submandibular oral/cutaneous fistula resulting in oral incompetence and leakage of food through a fistula while eating. Additionally, he had difficulties eating solid food and speaking. In January 2019, he underwent surgery, which consisted of a resection of mandible from angle to angle, reconstruction with a FFF, and temporary tracheostomy. (Figure 2B).

After reconstruction of the surgical defect with a fibula free flap, oral competence was restored and the patient was satisfied with the result. Because the original mandible was resected from angle to angle, his replacement mandible remained edentulous. This led to difficulties eating solid food, and the decreased ability to be understood while speaking. Eight months after his FFF reconstruction, dental implants were placed. Initially, osteosynthesis plates and screws were removed, and extra short implants (Bicon LLC, Boston, MA, USA) were placed at sites 43, 33, 46, and 36. All implants were placed subcrestally, except at site 36, where an implant was placed crestally. A vestibuloplasty was performed from sites 44 to 34. After four months of healing, the implants were uncovered and the abutments were placed. A free palatal mucosa graft was placed at site 33. One year after the implants were placed, a fiber-reinforced hybrid resin TRINIA^®^ (Bicon LLC, Boston, MA, USA) was fabricated (Figure 2C,D). After 40 months of function, the patient demonstrated very good compliance. He was eating solid food and was fully satisfied with his quality of life. The one dental implant that was placed crestally was stable and fully functional, without discomfort or mobility, despite having lost bone distally. These facts confirm Ewer’s findings that implants placed subcrestally showed no significant difference between the baseline bone level of (1.91 mm) and last follow up bone level of (2.12 mm). Implants placed supracrestally demonstrated a significant reduction of their bone levels over time (initial: 1.97 mm/final: 1.33 mm) [9].

### 2.2. Patient 2—Case Report of Extra Short Dental Implants in DCIA

**Figure 3:**
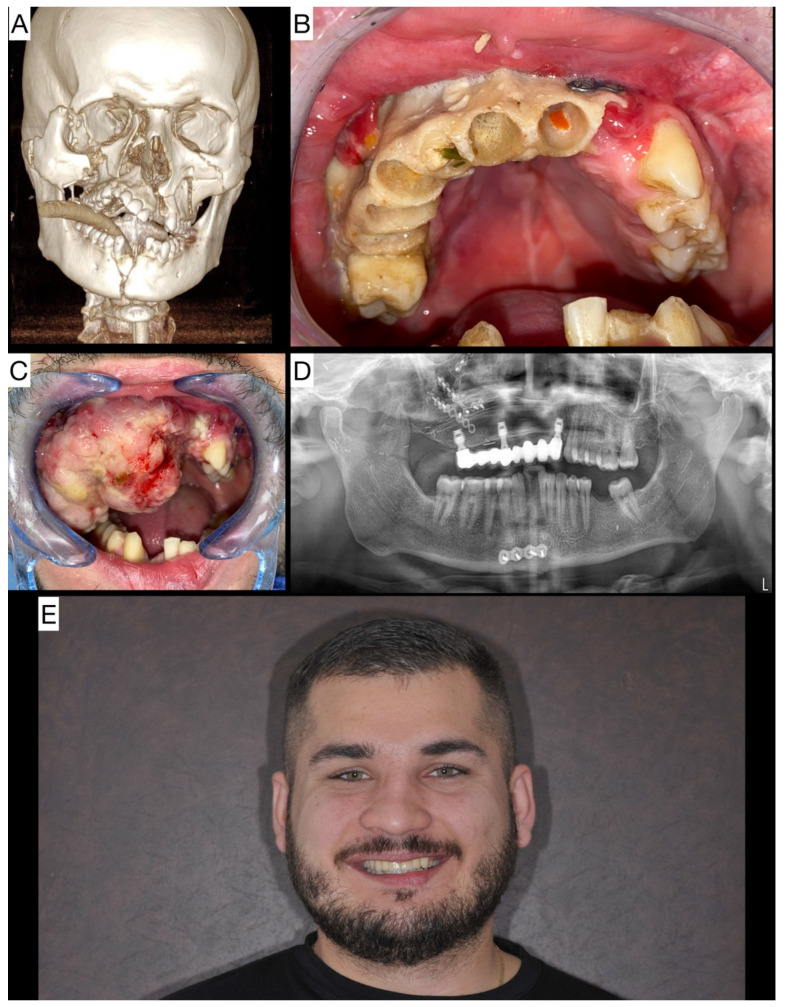
(**A**) 3D CT of panfacial trauma after a car accident. (**B**) Intraoral image of aseptic osteonecrosis of the maxilla, with demarcation of large bone segment from the surrounding healthy tissue. (**C**) Intraoral image after reconstruction of maxilla with DCIA and hyperplastic granulation of muscle tissue from the free flap. (**D**) OPG of final prosthesis supported by three extra short implants. (**E**) Image of the patient while smiling.

The second patient was a 26 year old man who had been injured on a car accident. The patient underwent CT polytrauma protocol, which revealed panfacial trauma of the maxilla and mandible with multiple fracture lines, described as Le Fort III *l.dx*., Le Fort II *l.sin*., and a dislocated fracture of the mandibular symphysis (Figure 3A). The patient underwent emergency osteosynthesis repositioning surgery. During the postoperative treatment, an aseptic osteonecrosis of right half of maxilla and premaxilla developed, with loss of dentition and demarcation of a large bone segment from the surrounding healthy bone (Figure 3B). Eleven months after the accident, the necrotic maxilla was resected, and a DCIA flap was harvested to reconstruct the defect. During his postoperative recovery, hyperplastic granulation tissue from the muscular tissue of the DCIA free flap was reduced several times. The anterior maxilla was augmented with bone from the hard palate (Figure 3C). Twenty months after panfacial trauma, some of the plates were removed from the maxilla, and three extra short Bicon dental implants (Bicon LLC) were placed. A metal ceramic prosthesis supported by three implants was fabricated, providing excellent aesthetics and function as well as a very satisfied patient (Figure 3D,E).

The survival and successful outcomes of the implants were evaluated using Kaplan–Meier (K-M) survival analyses (lifelines version 0.26.0, Python). Survival was defined as the implant or prosthesis remaining in situ throughout the duration of the study [30,31,32], while success was defined by the implant or prosthesis being in situ without complication or modification. Log-rank tests were performed to test for differences in K-M outcomes across different study parameters. The study was adherent to the STROBE (Strengthening the Reporting of Observational Studies in Epidemiology) guidelines checklist as seen in the Supplementary File.

## 3. Results

The results are summarized in Table 1, which is a list of patients with implants placed in OMFF. The longest follow-up period was 40 months, and the shortest period was seven months; the average was 28 months. Five of the patients have received their final prosthesis. No implants were lost during the clinical and radiological follow up.

**Figure 4:**
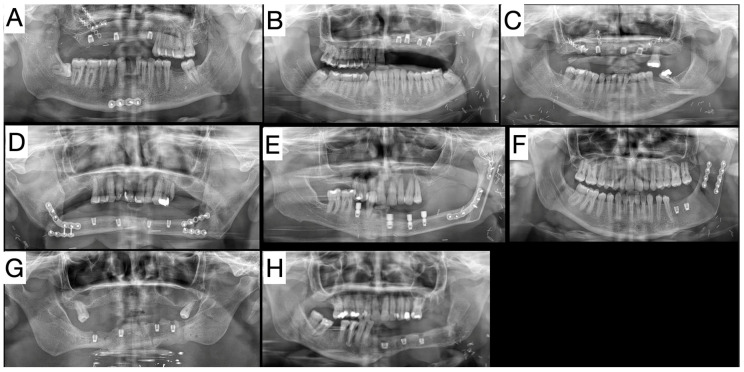
OPGs of our group of patients with short implants in free flap. Group consists of six patients with fibula free flap, and two patients with DCIA free flap. (**A**) Patient received reconstruction of maxilla on the right after aseptic traumatic necrosis caused by a car accident. We reconstructed the maxilla by DCIA free flap, patient is now prosthetically restored. (**B**) Young patient after hemimaxilectomy, with primary diagnosis adenoid cystic carcinoma. He first had his maxilla reconstructed with a DCIA free flap, which in this case failed, so we reconstructed it with fibula free flap. Patient is now prosthetically restored. (**C**) Patient after maxillectomy, with primary diagnosis of squamous cell carcinoma, reconstructed with a fibula free flap, also fully prosthetically restored. (**D**) Our first patient with extra short dental implants; because of osteoradionecrosis, we reconstructed the mandible with a fibula-free flap. He has had his dental prosthesis for almost five years, supported by extra short dental implants. (**E**) Patient after hemimandibulectomy, reconstructed with a fibula-free flap. Primary diagnosis was ameloblastoma associated with hypercalcemia. He is under prosthetic rehabilitation. (**F**) Our youngest patient, 21 year old female with a body and angle of mandible reconstructed with a DCIA free flap, with primary diagnosis of giant cell tumor. The patient is prosthetically restored. (**G**) A challenging case restored with extra short dental implants, after traumatic gunshot wound injury of the lower face caused by a suicide attempt; the mandible was reconstructed with a fibula-free flap, and the patient will receive prosthetic work in the near future. (**H**) Hemimandibulectomy reconstructed with a fibula-free flap, with a primary diagnosis of mucoepidermoid carcinoma. This patient received temporary prosthetic work, and is waiting for the final result.

This study investigated the survival and successful outcomes of 27 implants that were followed up for up to 40 months after implant surgery (Figure 4).

**Figure 5:**
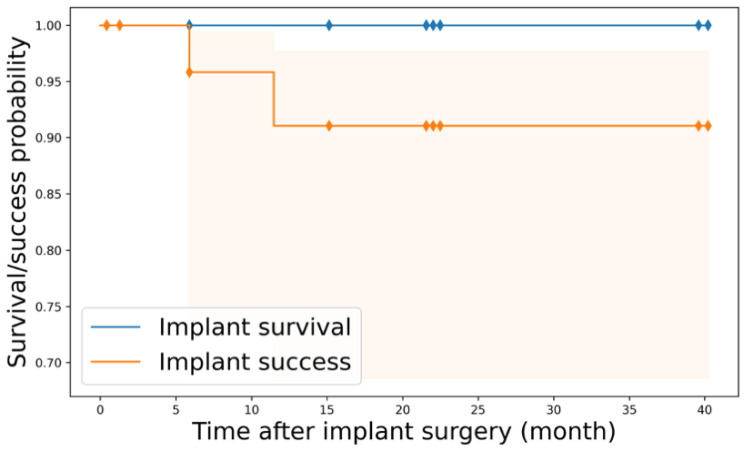
Kaplan–Meier survival curves for implant survival and success outcomes. Shaded regions represent 95% confidence intervals.

Kaplan–Meier (K-M) analyses revealed that the implant survival probability at 47 months was 100% (95% confidence interval: 100%), while the implant success probability was 91.7% (95% confidence interval: 70.7–97.8%) The K-M survival curves for implants are plotted in Figure 5.

Survival tables are reported in Table 2 and Table 3. Log-rank tests did not reveal any significant differences in implant success rates when correlated with any of the covariates investigated in this study, which included patient age, patient gender, FFF versus DCIA, implant location, implant dimensions, and the presence of grafting procedures.

Over the course of the study, two extra short dental implants developed peri-implantitis, but remained in situ throughout the study period. Five finished prostheses were installed, with neither failures nor complications during the follow-up period of 40 months after their insertion. The K-M survival and success rates for the prostheses were 100% at 35 months (95% confidence interval: 100%)

## 4. Discussion

Although placing dental implants in microvascular free bone flaps is standard treatment, placing short and extra short dental implants in OMFF has not been attempted until recently. The literature [33] regarding short dental implants placed in OMFF is scarce. The volume of bone available in microvascular free flaps is less than that of native mandibular and maxillary structures. Placing conventional dental implants of standard diameters and lengths often requires additional bone augmentation with predictable increases in treatment morbidity, time, and cost [34,35,36]. It has been reported that clinicians must emphasize to patients and caregivers to provide customized patient prosthetic accessibility for oral hygiene procedures [37].

In all situations, maintenance of bone levels around implants is of paramount importance. This is especially true for threaded short and/or extra-short implants [38]. Therefore, we have opted for plateau-root form implants because their macrogeometry provides for different clinical capabilities [39]. Also, direct bone formation occurs at the osteotomy due to their unique osseointegration healing pathway that rapidly evolves towards formation of a Haversian-like bone morphology with high mechanical properties. The process of unique bone formations for plateau-root form implants has been extensively described [40,41,42,43,44]. Also, crestal bone loss in FFF grafts has been reported to be on average 2.0 mm [45]. Improved results may likely be expected due the locking taper design at the abutment/implant interface. This has previously been shown to provide an aseptic impermeable seal [46].

Based on the present case series, extra short implants served as a promising solution for placement in osseous microvascular free flaps. Such implants are less invasive and have high survivability, but longer follow-up times are needed [47]. Following implant placement, it is imperative that prosthetic rehabilitation be initiated as soon as possible to secure improvements in the patients’ speech and mastication, and to assure their overall quality of life.

As mentioned for patients with OMFFs, we try to avoid further surgical procedures due to a fundamental concern minimizing a patient’s exposure to additional morbidities. Short dental implants have the unmistakable advantage of size, which most often precludes the need for augmentation surgeries and their associated morbidity [48,49]. According to Malet, the most important clinical recommendations for placing short implants [50] is to avoid generating heat. The placement protocol for the implants used in this study require operating at 50 rpm (rounds per minute) or less, without cooling; this supports Malet’s recommendation. These authors do not recommend immediate implant placement nor immediate loading. The best time to place implants in OMFF is still a matter of debate. Operating at low speeds and without cooling, the system used in this study is also less traumatic, resulting in preservation of the vascular pedicle and the vascular supply of the bone free flap [51]. Also, during the preparation of the osteotomy, the harvested bone, being identical with the fibula or DCIA bone (iliac crest), can be used as an augmentation material around the implant.

Considering the fact that the expected bone loss in OMFF is greater than in native bone [45], it is essential that these short and extra short dental implants be placed subcrestally in free bone flaps as prescribed. In our cohort of 27 short and extra short implants, bone recession was observed at one implant site where the implant was placed crestally instead of subcrestally. Although our results are promising, we acknowledge that our sample size is small and that is a limitation in this study. More studies with a larger sample size are warranted to corroborate the use of extra-short and short implants in the rehabilitation of OMFF.

## 5. Conclusions

Short and extra short dental implants placed in osseous microvascular free flaps presented high survival and success rates in this study with up to 40 months follow-up. Longer follow-up times and a larger sample size are warranted.

## Figures and Tables

**Table 1:** List of patients with short and extra short dental implants placed in microvascular free flaps.

	Sex	Age at Time of Placing Dental Implants	Reconstructed Jaw	Type of Free Flap *	Number of Dental Implants in Free Flap	Implant Size (Bicon, Bicon LLC)	Status
Patient No. 1	Male	67	Mandible	FFF	4	4.0 × 6.0 Integra CP	Prosthetic work in function
Patient No. 2	Male	51	Mandible	FFF	3	4.0 × 6.0 Integra CP	Adjusting prosthetic work
Patient No. 3	Female	55	Maxilla	FFF	4	4.0 × 6.5 Integra CP	Prosthetic work in function
Patient No. 4	Male	24	Maxilla	FFF	4	4.5 × 6.0 Integra CP (2×) 4.5 × 8.0 Integra CP (2×)	Prosthetic work in function
Patient No. 5	Male	26	Maxilla	DCIA	3	4.5 × 6.0 Integra CP (1×) 5.0 × 6.0 Integra CP (2×)	Prosthetic work in function
Patient No. 6	Female	21	Mandible	DCIA	2	4.5 × 6.0 Integra CP (1×) 5.0 × 6.0 Integra CP (1×)	Prosthetic work in function
Patient No. 7	Male	46	Mandible	FFF	4	5.0 × 6.0 Integra CP (4×)	Adjusting prosthetic work
Patient No. 8	Female	55	Mandible	FFF	3	4.5 × 5.0 mm Integra-CP (3×)	Loaded interim fixed prostheses

* FFF—fibula free flap, DCIA—deep circumflex iliac artery flap, calcium phosphate—CP, dental implant—DI.

**Table 2:** Kaplan–Meier survival table for implant survival rates.

Implants at Risk	Time (Months)				
	0	10	20	30	40
At risk	27	27	24	20	7
Censored	0	3	4	13	0
Implant failure	0	0	0	0	0
Implant survival probability	1.00	1.00	1.00	1.00	1.00

**Table 3:** Kaplan–Meier survival table for implant success rates.

Implants at Risk	Time (Months)				
	0	10	20	30	40
At risk	27	27	24	19	6
Censored	0	3	3	13	0
Implant complication	0	0	2	0	0
Implant success probability	1.00	1.00	0.917	0.917	0.917

## Data Availability

Research data regarding this manuscript can be directly obtained from first and senior authors upon request.

## Supplementary Materials

The following supporting information can be downloaded at: https://www.mdpi.com/article/10.3390/jpm14040384/s1.
