# Supplementary material for: Short and Extra Short Dental Implants in Osseous Microvascular Free Flaps: A Retrospective Case Series
Source: J Pers Med. 2024 Apr 3;14(4):384. doi: 10.3390/jpm14040384 (PMC11050822; doi:10.3390/jpm14040384)
Supplement: Supplementary file 1 [file jpm-14-00384-s001.zip › jpm-2910109-supplementary.pdf]

STROBE Statement—Checklist of items that should be included in reports of ***cross-sectional studies***

	Item No	Recommendation
**Title and abstract**	1	(*a*) Indicate the study's design with a commonly used term in the title or the abstract p1,3
		(*b*) Provide in the abstract an informative and balanced summary of what was done and what was found p3
**Introduction**		
Background/rationale	2	Explain the scientific background and rationale for the investigation being reported p4-5
Objectives	3	State specific objectives, including any prespecified hypotheses p5
**Methods**		
Study design	4	Present key elements of study design early in the paper p3
Setting	5	Describe the setting, locations, and relevant dates, including periods of recruitment, exposure, follow-up, and data collection p6
Participants	6	(*a*) Give the eligibility criteria, and the sources and methods of selection of participants p6-7
Variables	7	Clearly define all outcomes, exposures, predictors, potential confounders, and effect modifiers. Give diagnostic criteria, if applicable p6-7
Data sources/ measurement	8*	For each variable of interest, give sources of data and details of methods of assessment (measurement). Describe comparability of assessment methods if there is more than one group p7-8
Bias	9	Describe any efforts to address potential sources of bias na
Study size	10	Explain how the study size was arrived at na
Quantitative variables	11	Explain how quantitative variables were handled in the analyses. If applicable, describe which groupings were chosen and why p7
Statistical methods	12	(*a*) Describe all statistical methods, including those used to control for confounding p7
		(*b*) Describe any methods used to examine subgroups and interactions p7
		(*c*) Explain how missing data were addressed na
		(*d*) If applicable, describe analytical methods taking account of sampling strategy na
		(*e*) Describe any sensitivity analyses na
**Results**		
Participants	13*	(a) Report numbers of individuals at each stage of study—eg numbers potentially eligible, examined for eligibility, confirmed eligible, included in the study, completing follow-up, and analysed p7-8
		(b) Give reasons for non-participation at each stage na
		(c) Consider use of a flow diagram na
Descriptive data	14*	(a) Give characteristics of study participants (eg demographic, clinical, social) and information on exposures and potential confounders P7-8
		(b) Indicate number of participants with missing data for each variable of interest p7-8
Outcome data	15*	Report numbers of outcome events or summary measures p7-10
Main results	16	(*a*) Give unadjusted estimates and, if applicable, confounder-adjusted estimates and their precision (eg, 95% confidence interval). Make clear which confounders were adjusted for and why they were included na
		(*b*) Report category boundaries when continuous variables were categorized na

		(*c*) If relevant, consider translating estimates of relative risk into absolute risk for a meaningful time period na
Other analyses	17	Report other analyses done—eg analyses of subgroups and interactions, and sensitivity analyses na
**Discussion**		
Key results	18	Summarise key results with reference to study objectives p13
Limitations	19	Discuss limitations of the study, taking into account sources of potential bias or imprecision. Discuss both direction and magnitude of any potential bias p13
Interpretation	20	Give a cautious overall interpretation of results considering objectives, limitations, multiplicity of analyses, results from similar studies, and other relevant evidence p13
Generalisability	21	Discuss the generalisability (external validity) of the study results p13
**Other information**		
Funding	22	Give the source of funding and the role of the funders for the present study and, if applicable, for the original study on which the present article is based p2

*Give information separately for exposed and unexposed groups.

Note: An Explanation and Elaboration article discusses each checklist item and gives methodological background and published examples of transparent reporting. The STROBE checklist is best used in conjunction with this article (freely available on the Web sites of PLoS Medicine at http://www.plosmedicine.org/, Annals of Internal Medicine at http://www.annals.org/, and Epidemiology at http://www.epidem.com/). Information on the STROBE Initiative is available at www.strobe-statement.org.
